# The TRIAGE-ProADM Score for an Early Risk Stratification of Medical Patients in the Emergency Department - Development Based on a Multi-National, Prospective, Observational Study

**DOI:** 10.1371/journal.pone.0168076

**Published:** 2016-12-22

**Authors:** Alexander Kutz, Pierre Hausfater, Devendra Amin, Adina Amin, Pauline Canavaggio, Gabrielle Sauvin, Maguy Bernard, Antoinette Conca, Sebastian Haubitz, Tristan Struja, Andreas Huber, Beat Mueller, Philipp Schuetz, for the TRIAGE study group

**Affiliations:** 1 Division of General and Emergency Medicine; University Department of Medicine, Kantonsspital Aarau, Aarau, Switzerland; 2 Emergency Department, Groupe Hospitalier Pitié-Salpêtrière, Assistance Publique-Hôpitaux de Paris (APHP), Paris, France; 3 Sorbonne Universités UPMC-Univ Paris06, UMRS INSERM 1166, IHUC ICAN, Paris, France; 4 Morton Plant Hospital, Clearwater, FL, United States of America; 5 Biochemistry Department, Hôpital Pitié-Salpêtrière and Univ-Paris Descartes, Paris, France; 6 Department of Laboratory Medicine, Kantonsspital Aarau, Aarau, Switzerland; Universitatsklinikum Hamburg-Eppendorf, GERMANY

## Abstract

**Introduction:**

The inflammatory biomarker pro-adrenomedullin (ProADM) provides additional prognostic information for the risk stratification of general medical emergency department (ED) patients. The aim of this analysis was to develop a triage algorithm for improved prognostication and later use in an interventional trial.

**Methods:**

We used data from the multi-national, prospective, observational TRIAGE trial including consecutive medical ED patients from Switzerland, France and the United States. We investigated triage effects when adding ProADM at two established cut-offs to a five-level ED triage score with respect to adverse clinical outcome.

**Results:**

Mortality in the 6586 ED patients showed a step-wise, 25-fold increase from 0.6% to 4.5% and 15.4%, respectively, at the two ProADM cut-offs (≤0.75nmol/L, >0.75–1.5nmol/L, >1.5nmol/L, p ANOVA <0.0001). Risk stratification by combining ProADM within cut-off groups and the triage score resulted in the identification of 1662 patients (25.2% of the population) at a very low risk of mortality (0.3%, n = 5) and 425 patients (6.5% of the population) at very high risk of mortality (19.3%, n = 82). Risk estimation by using ProADM and the triage score from a logistic regression model allowed for a more accurate risk estimation in the whole population with a classification of 3255 patients (49.4% of the population) in the low risk group (0.3% mortality, n = 9) and 1673 (25.4% of the population) in the high-risk group (15.1% mortality, n = 252).

**Conclusions:**

Within this large international multicenter study, a combined triage score based on ProADM and established triage scores allowed a more accurate mortality risk discrimination. The TRIAGE-ProADM score improved identification of both patients at the highest risk of mortality who may benefit from early therapeutic interventions (rule in), and low risk patients where deferred treatment without negatively affecting outcome may be possible (rule out).

## Introduction

Timely and an accurate risk stratification of patients in the emergency department (ED) supports the clinician in reducing time to effective treatment—a key predictor for patient outcome. A shorter time to effective treatment is an important outcome predictor for patients with septicemia [1], pneumonia [2], stroke (“time is brain”) [3], and myocardial infarction (“time is heart”) [4]. Yet, due to increasingly patient (over-) crowding in the ED, an early identification of patients requiring urgent care is challenging, resulting in a delayed time to medication and possibly poor health outcomes [5, 6]. The use of accurate triage tools early in the process of ED admission has the potential to identify high risk patients and at the same time rule out risk in other patients that may not clinically benefit from urgent care. For several patient populations, international guidelines recommend the use of risk scores for this very purpose such as the pneumonia severity index (PSI) or the CURB-65 score in community-acquired pneumonia (CAP) patients [7, 8]. Yet, there is a lack of a more general risk stratification score for medical patients at the most proximal time point of ED care when no final diagnosis has yet been established. At this early and crucial time in patient care, risk stratification is most challenging due to a lack of relevant clinical information, but may indeed have the largest impact to improve timeliness of ED care, and thus, patient flow and outcome.

Accordingly, several triage scores in the ED have been consequently proposed [9, 10]. These scores assign patients based on their presenting symptoms and a combination of vital signs into risk categories with different recommended times for the initial physician assessment [9]. The main rational of these scores is to stratify treatment urgency based on clinical symptoms (“red flags”). As a limitation, only few rigorous clinical studies have investigated the performance of initial triage scores for their ability to improve initial triage decisions and patient outcomes [9, 11]. In addition to clinical-parameter based triage scores, there is increasing interest in the use of prognostic blood biomarkers from different pathophysiological pathways that may add prognostic information [12]. The recent TRIAGE trial found a high prognostic ability of the inflammatory marker pro-adrenomedullin (ProADM) for the prediction of 30-day mortality, intensive care unit (ICU) admission, and high initial treatment priority [13, 14]. ProADM was also found to be of a high prognostic utility in several other studies in patients with respiratory tract infections and sepsis [15–21]. Physiologically, ProADM reveals natriuretic and vasodilatatory effects, and is expressed in different tissues, where it acts both as an autocrine and paracrine mediator [22, 23]. ProADM is also a marker for hemodynamic status and cardiovascular dysfunction, and is highly predictive for adverse outcomes in patients with heart failure [24]. Indeed, ProADM levels are associated with important cardiovascular risk factors in elderly patients [25], and predict mortality in the general population [13, 26]. Moreover, in unselected patients presenting to an emergency department, ProADM plasma levels are elevated in proportion to disease severity, and possess the unique ability to identify patients at risk for short-term mortality beyond clinical risk assessment [27].

The aim of this analysis was to develop a clinical algorithm based on a combination of a clinical triage score and ProADM levels for an improved prognostic assessment in unselected medical ED patients. The effects of using these scores on timeliness and patient outcomes will later be investigated in an intervention trial.

## Material and Methods

### Study design

The TRIAGE study is a multi-national, prospective, observational cohort study. From March 2013 to October 2014, we included consecutive medical patients presenting with a medical urgency at three participating tertiary care hospitals in Aarau (Switzerland), Paris (France), and Clearwater (Florida, USA), respectively. As an observational quality control study, the Institutional Review Boards (IRB) of the three hospitals approved the study and waived the need for individual informed consent (main Swiss IRB: Ethikkommission Kanton Aargau (EK 2012/059); French IRB: CCTIRS—Le Comité consultatif sur le traitement de l'information en matière de recherche (C.C.T.I.R.S.) (CPP ID RCB: 2013-A00129-36); US IRB MPM-SAH Institutional Review Board, Clearwater Florida [IRB number 2013_005]).

The study was registered at the “ClinicalTrials.gov” registration website (http://www.clinicaltrials.gov/ct2/show/NCT01768494) and the study protocol, as well as more detailed information about the study design, has been published previously [13, 28].

### Patient sample

All patients seeking ED care for medical health issues and meeting our inclusion criteria at one of the participating hospital EDs were consecutively included. Inclusion criteria were adult medical patients in whom an initial blood draw was done as part of the routine ED assessment. We excluded surgical and pediatric patients, but had no other exclusion criteria with regard to main medical disciplines or presenting symptoms to reflect the diversity and challenges of “real-life”.

### Data collection

Upon ED admission, all patients were assessed by a triage nurse and initial triage priority (five-level system; “blue”, very low urgency; “green”, low urgency; “yellow”, intermediate urgency; “orange”, high urgency; “red”, very high urgency) was assigned based on the routine hospital algorithm [9]. All participants provided a thorough medical history and underwent a physical examination with measurement of vital signs and laboratory assessment with collection of left over blood samples. We also recorded main presenting clinical symptoms and complaints, socio-demographics and comorbidities. Upon ED discharge, two independent attending ED physicians adjudicated a medical triage priority post hoc based on all medical results available at this time to all patients (low vs. high initial treatment priority). In case of disagreement, the case was discussed with a third independent physician until consensus was reached. All information was entered into a case report form and stored in a centralized, password secured databank (SecuTrial).

Throughout the hospital stay, physicians, nurses and social care workers managed patients in accordance to local hospital guidelines according to the underlying medical condition and independently of the research team. All patients were contacted by telephone interview 30 days after hospital admission using a predefined questionnaire to assess vital and functional status, and unplanned hospital readmission among other outcomes.

### Overall hypothesis and research aim

The main research aim of this secondary analysis was to derive a triage algorithm based on ProADM and triage risk classes using already established ED triage scores for an improved prognostic assessment in unselected medical ED patients. ProADM was first used within cut-off ranges based on previous studies [20] and in a later step as a continuous variable.

### Study endpoints

In accordance with the initial study, our primary endpoint was 30-day mortality following ED admission. To verify survival status, we followed all patients during hospital stay and contacted them 30 days after inclusion by telephone.

### Blood draws and ProADM measurement

Left over blood samples of routinely collect blood tubes on admission were immediately centrifuged, aliquoted and frozen at -20° C for later batch analysis of blood biomarkers. The results of these analyses were not available at the time of hospitalization of the patients. Thus, physicians, patients and outcome adjudicators were blinded to their results. ProADM was batch-measured with a sandwich immunoassay with an analytical detection limit of 0.08nmol/L as described elsewhere [29].

### Statistical analysis

We used descriptive statistics including mean with standard deviation (SD), median with interquartile range (IQR) and frequencies to describe the populations, as appropriate. We displayed observed and expected probabilities for all adverse outcomes within deciles of ProADM and goodness-of-fit assessed with the Hosmer-Lemeshow test. Based on these analyses and previous research [20], we defined two ProADM cut-off levels which separated patients into low (≤0.75nmol/L), intermediate (>0.75–1.5nmol/L), and high levels (>1.5nmol/L). Using these two ProADM cut-off values, we firstly risk-stratified patients, and secondly investigated the clinical usefulness of these ProADM cut-offs when combined with the ED triage score by calculating observed risk for 30-day mortality. For this purpose, “blue” and “green” patients (according to the triage score) were labeled as “not urgent”, “yellow” patients were labeled as “moderate urgent”, and “orange” and “red” patients as “very urgent”. Furthermore, we designed a clinical algorithm based on the ED triage score, at which “not urgent (“blue”, “green”)”, “moderate urgent (“yellow”)” and “very urgent (“orange”, “red”)” patients were reclassified by combining with ProADM cut-off levels. In doing so, formerly “not urgent” or “moderate urgent” patients with ProADM values >1.5nmol/L were reclassified to the next higher risk category, however “moderate urgent” and “very urgent” patients with ProADM levels ≤0.75nmol/L were reclassified to the next lower category. Using net reclassification analysis (NRI), we calculated the effect of combining the triage score and ProADM. To more accurately address confounders in 30-day mortality risk prediction, we calculated adjusted (age, gender, main symptoms on admission, and main medical disciplines as additional fixed effects) odds ratios (OR) using multivariable logistic regression. The recalibrated risk for mortality can be calculated as 1/(1+exp(-(α+β*log (risk/(1-risk))))). Intercept α, calibration slope β, and odds are displayed in the supporting information. β = (β1, …, βn).

Tests were two-tailed and carried out at 5% significance levels. Analyses were performed with STATA 12.1 (Stata Corp., College Station, TX, USA).

## Results

### Patient population

From a total of 7342 ED patients initially included in participating Swiss, French, and United States (USA) hospitals, 6586 patients (53.3% males, median age 62 years) had complete follow-up information, as well as biomarker and triage score data and were included in the final analysis (n = 4103, n = 1489, n = 994, respectively). The most prevalent principal diagnoses were cardiovascular diseases (25.4%), neurological diseases (20.9%), gastrointestinal diseases (13.2%), and acute infections (13.1%). We observed a high prevalence of comorbidities including hypertension, coronary heart disease, diabetes, and cancer. Regarding adverse outcomes within 30 days of ED admission, 4.8% of patients died and 6.8% of patients were admitted to ICU. Additional patient characteristics are shown in **Table 1**. Overall ProADM distribution is skewed right as shown by a histogram (**S1 Fig**).

**Table 1:** Patient characteristics.

	ProADM categories			
	ProADM ≤0.75nmol/L	ProADM >0.75–1.5nmol/L	ProADM >1.5nmol/L	p-value
	n = 2972	n = 2382	n = 1232	
**Socio-demographics**				
Age, median (IQR)	49 (35, 61)	71 (60, 80)	76 (66, 84)	<0.001
Male Gender, n (%)	1562 (52.6%)	1251 (52.5%)	703 (57.1%)	0.017
**Vital signs**				
Respiratory rate, median (IQR)	18 (18, 20)	18 (18, 20)	18 (18, 22)	0.005
SO2, median (IQR)	97 (96, 99)	96 (94, 98)	95 (92, 97)	<0.001
Confusion, n (%)	136 (4.6%)	206 (8.6%)	137 (11.1%)	<0.001
BPS, median (IQR)	138 (124, 153)	141 (123, 159)	127 (109, 147.5)	<0.001
BPD, median (IQR)	84 (75, 93)	79 (70, 90)	71 (60, 82)	<0.001
PR, median (IQR)	81.75 (70, 94)	84 (71, 98)	87 (72, 103)	<0.001
Temperature, °C, median (IQR)	36.8 (36.4, 37.1)	36.8 (36.5, 37.3)	36.9 (36.4, 37.6)	<0.001
**Main symptoms at ED admission, n (%)**				
Non-thoracic pain	706 (23.8%)	297 (12.5%)	110 (8.9%)	<0.001
Thoracic pain	619 (20.8%)	320 (13.4%)	58 (4.7%)	
Neurological symptoms	584 (19.7%)	506 (21.2%)	134 (10.9%)	
Respiratory Symptoms	247 (8.3%)	367 (15.4%)	321 (26.1%)	
General worsening	268 (9.0%)	321 (13.5%)	242 (19.6%)	
Blood loss	50 (1.7%)	75 (3.1%)	72 (5.8%)	
Diarrhea, vomitus, dysuria	196 (6.6%)	179 (7.5%)	114 (9.3%)	
Fever	75 (2.5%)	138 (5.8%)	124 (10.1%)	
Other	227 (7.6%)	179 (7.5%)	57 (4.6%)	
**Main medical disciplines, n (%)**				
Infection	265 (8.9%)	312 (13.1%)	288 (23.4%)	<0.001
Cardiovascular	775 (26.1%)	595 (25.0%)	301 (24.4%)	
Metabolic	102 (3.4%)	53 (2.2%)	79 (6.4%)	
Cancer	47 (1.6%)	133 (5.6%)	107 (8.7%)	
Neurological	749 (25.2%)	527 (22.1%)	99 (8.0%)	
Gastrointestinal	420 (14.1%)	301 (12.6%)	150 (12.2%)	
Pulmonary	164 (5.5%)	199 (8.4%)	106 (8.6%)	
Other	450 (15.1%)	262 (11.0%)	102 (8.3%)	
**Co-morbidities, n (%)**				
Hypertension	766 (25.8%)	1289 (54.1%)	634 (51.5%)	<0.001
Congestive heart failure	40 (1.3%)	168 (7.1%)	274 (22.2%)	<0.001
Coronary artery disease	254 (8.5%)	358 (15.0%)	197 (16.0%)	<0.001
COPD	76 (2.6%)	175 (7.3%)	100 (8.1%)	<0.001
Dementia	17 (0.6%)	124 (5.2%)	78 (6.3%)	<0.001
Diabetes	249 (8.4%)	458 (19.2%)	353 (28.7%)	<0.001
History of stroke	199 (6.7%)	271 (11.4%)	73 (5.9%)	<0.001
Substance abuse	223 (7.5%)	143 (6.0%)	73 (5.9%)	0.047
Cancer	215 (7.2%)	414 (17.4%)	303 (24.6%)	<0.001
Chronic renal failure	51 (1.7%)	237 (9.9%)	552 (44.8%)	<0.001
**Initial blood biomarkers, median (IQR)**				
Hemoglobin [g/L]	14.2 (13.1, 15.2)	13.3 (12.0, 14.5)	11.5 (9.8, 13.1)	<0.001
WBC [10^9^/L]	7.9 (6.3, 9.9)	8.58 (6.67, 11.27)	9.74 (7.11, 13.5)	<0.001
Sodium [mmol/L]	139 (138, 141)	138 (136, 140)	137 (134, 140)	<0.001
Glucose [mmol/L]	5.8 (5.2, 6.7)	6.4 (5.5, 7.7)	7.0 (5.8, 8.9)	<0.001
Creatinine [μmol/L]	71.0 (60.0, 83.0)	85.0 (71.0, 103.0)	139.5 (103.0, 210.5)	<0.001
C-reactive protein [mg/L]	.9 (<3, 7)	8.7 (<3, 41.7)	40.6 (10.5, 122)	<0.001
ProADM [nmol/L]	0.6 (0.5, 0.6)	1.0 (0.8, 1.2)	2.2 (1.8, 3.3)	<0.001
**Initial triage score, n (%)**				
No emergency	926 (31.2)	403 (16.9%)	68 (5.5%)	<0.001
Within 90 minutes	736 (24.8%)	375 (15.7%)	174 (14.1%)	
Within 30 minutes	815 (27.4%)	921 (38.7%)	565 (45.9%)	
Within 10 minutes	375 (12.6%)	525 (22.0%)	320 (26.0%)	
Immediate treatment needed	120 (4.0%)	158 (6.6%)	105 (8.5%)	
**Patient outcomes, n (%)**				
ICU admission	116 (3.9%)	153 (6.4%)	178 (14.4%)	<0.001
30-day mortality	19 (0.6%)	106 (4.5%)	190 (15.4%)	<0.001
Length of stay	4.8 (5.4)	6.0 (5.9)	7.9 (6.7)	<0.001

IQR, Interquartile range; SO2, oxygen saturation; BPS, blood pressure systolic; BPD, blood pressure diastolic; PR, pulse rate; ED, emergency department; COPD, chronic obstructive pulmonary disease; WBC, white blood cell count; ProADM, pro-adrenomedullin; ICU, intensive care unit.

### Definition of ProADM cut-off values for optimal risk prediction

**Fig 1:**
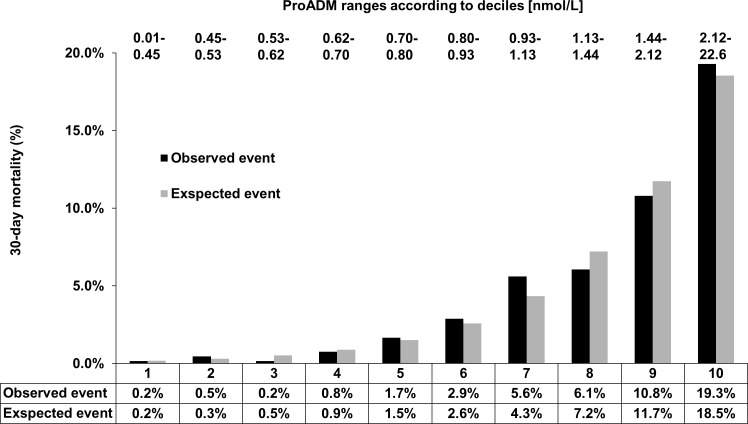
Definition of ProADM cut-off values for 30-day mortality prediction. p (goodness of fit) = 0.494; ProADM, pro-adrenomedullin.

As demonstrated in **Fig 1**, we estimated associations between admission ProADM levels divided into deciles and 30-day mortality. There was a very low observed mortality in the lowest four ProADM deciles (i.e., ≤0.75nmol/L) with an exponential increase thereafter, particularly in the highest 2 ProADM deciles (i.e., >1.5nmol/L). We therefore used ProADM cut-off levels of ≤0.75nmol/L indicating low risk, >0.75–1.5nmol/L indicating moderate risk, and >1.5nmol/L indicating high risk. The overall risk for 30-day mortality showed an almost 25-fold (from 0.6% to 4.5% and 15.4%) increase within these ProADM groups (p<0.0001, **Table 2**). For patients in the low risk ProADM group (i.e. <0.75nmol/L), mortality could be excluded with a high negative predictive value (NPV) of 99.4% (95% CI: 99.0 to 99.6) and a sensitivity of 94.0% (95% CI: 90.7 to 96.3). Conversely, in patients with high ProADM levels (i.e., >1.5nmol/L) specificity was 83.4% (95% CI: 82.4 to 84.3) and positive predictive value (PPV) was 15.4% (95% CI: 13.4 to 17.6) (**Table 3**).

**Table 2:** 30-day mortality according to predefined ProADM categories.

	ProADM categories						P value
Adverse event (95% CI)	n	ProADM ≤0.75nmol/L	n	ProADM >0.75–1.5nmol/L	n	ProADM >1.5nmol/L	
***Primary endpoint***							
30-day mortality	2972	0.6 (0.4–0.9)	2382	4.5 (3.6–5.3)	1232	15.4 (13.4–17.4)	<0.0001

ProADM, pro-adrenomedullin; CI, confidence interval.

**Table 3:** Mortality risk prediction according to predefined ProADM categories.

ProADM cut-offs (nmol/L)	0.75nmol/L				1.5nmol/L			
	Sens. (%) (95% CI)	Spec. (%) (95% CI)	PPV (%) (95% CI)	NPV (%) (95% CI)	Sens. (%) (95% CI)	Spec. (%) (95% CI)	PPV (%) (95% CI)	NPV (%) (95% CI)
***Primary endpoint***								
30-day mortality (overall)	94.0 (90.7, 96.3)	47.1 (45.8, 48.3)	8.2 (7.3, 9.1)	99.4 (99.0, 99.6)	60.3 (54.7, 65.8)	83.4 (82.4, 84.3)	15.4 (13.4, 17.6)	97.7 (97.2, 98.1)
30-day mortality (not urgent)	91.9 (82.2, 97.3)	63.2 (61.4, 65.1)	5.6 (4.3, 7.2)	99.7 (99.3, 99.9)	53.2 (40.1, 66.0)	92.0 (90.9, 93.0)	13.6 (9.6, 18.6)	98.8 (98.3, 99.2)
30-day mortality (moderate urgent)	94.3 (88.1, 97.9)	36.9 (34.8, 38.9)	6.7 (5.5, 8.1)	99.3 (98.4, 99.7)	70.8 (61.1, 79.2)	77.7 (75.9, 79.4)	13.3 (10.6, 16.4)	98.2 (97.5, 98.8)
30-day mortality (very urgent)	94.6 (89.6, 97.6)	33.4 (31.0, 35.9)	12.5 (10.7, 14.6)	98.4 (96.8, 99.3)	55.8 (47.4, 64.0)	76.4 (74.2, 78.6)	19.3 (15.6, 23.4)	94.5 (93.0, 95.7)

ProADM, pro-adrenomedullin; Sens., sensitivity; Spec., specificity; PPV, positive predictive value; NPV, negative predictive value; CI, confidence interval.

### Combination of ProADM and initial ED triage levels

**Fig 2:**
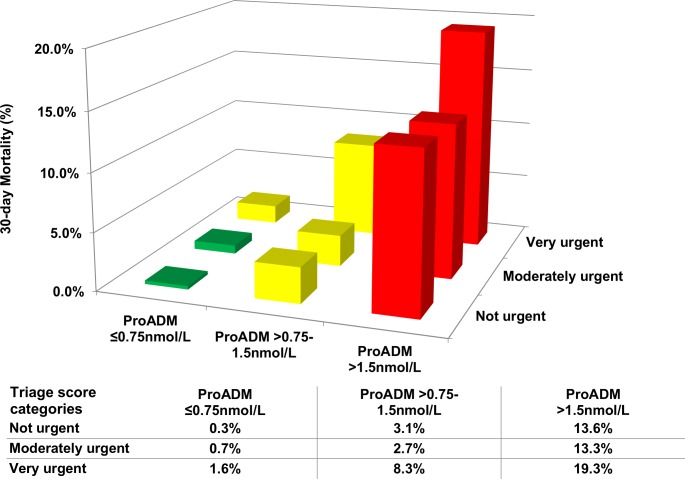
Observed risk assessment combining initial emergency department triage score information and ProADM cut-off values. ProADM, pro-adrenomedullin.

In a next step, we combined the three ProADM categories (≤0.75nmol/L, >0.75–1.5nmol/L, >1.5nmol/L) with triage score risk subgroups (“not urgent”, “moderate urgent”, “very urgent”). The addition of ProADM further improved prognostication of the triage score for all patient groups (**Fig 2**). The proportion of 30-day non-survivors increased from 0.3% in patients classified as “not urgent” and ProADM ≤0.75nmol/L to 19.3% in patients classified as “very urgent” and ProADM >1.5nmol/L. In patients classified as “not urgent” according to the triage score, we found a strong increase in the risk of 30-day mortality with increasing ProADM levels (from 0.3% to 13.6%). **Table 3** shows sensitivity, specificity and positive and negative predictive values at different triage score as well as ProADM cut-offs.

### Derivation of a clinical algorithm to improve ED triage

**Fig 3:**
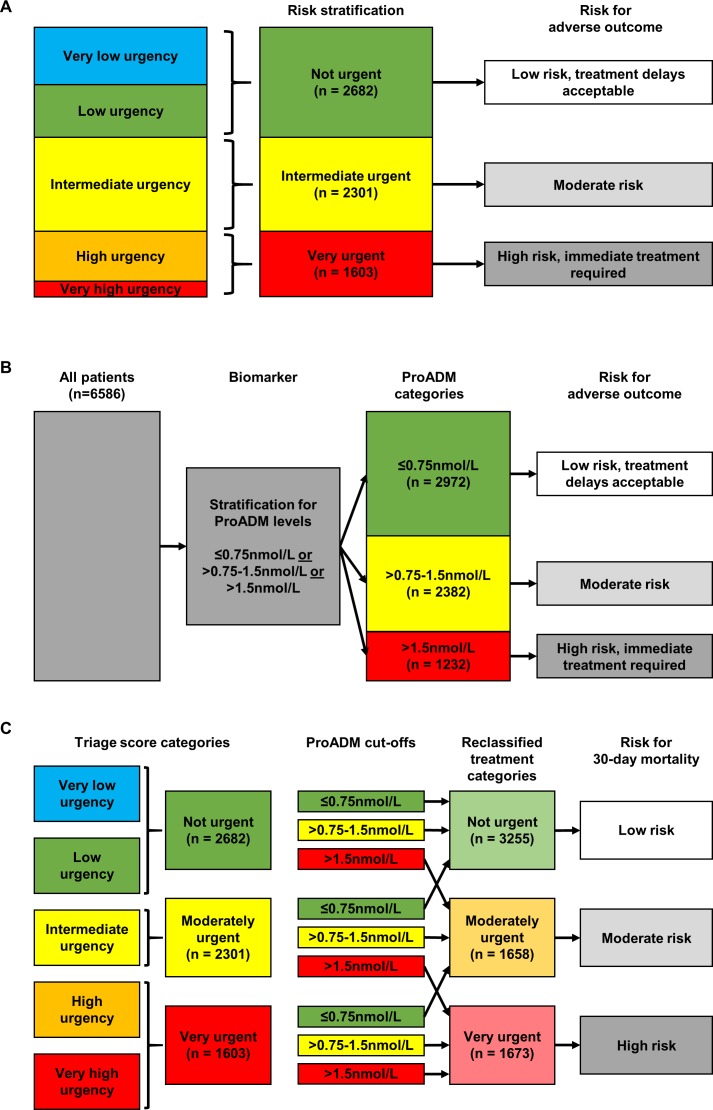
Derivation of a biomarker based algorithm combining the triage score and ProADM to more efficiently triage patients at risk for 30-day mortality. (A) Triage score based risk stratification, (B) ProADM based risk stratification, (C) Combined model. ProADM, pro-adrenomedullin.

Using the above analyses, we developed a simplified easy to use clinical algorithm based on the initial triage score and the ProADM category and used it virtually within our patient cohort. Based on the triage score, 21.2% (n = 1397) of patients were initially classified as very low urgent (“blue”), 19.5% (n = 1285) as low urgent (“green”), 34.9% (n = 2301) as intermediately urgent (“yellow”), 18.5% (n = 1220) as highly urgent (“orange”), and 5.8% (n = 383) as very urgent (“red”). These patients were further classified in three categories as shown in **Fig 3A** (not urgent [“blue”, “green”], moderate urgent [“yellow”], and very urgent [“orange”, “red”]). When only using ProADM to classify patients, 45.1% (n = 2972) of patients had biomarker levels ≤0.75nmol/L and had thus a low risk of adverse outcomes. A total of 36.2% (n = 2382) were at an intermediate risk (ProADM >0.75–1.5nmol/L) and 18.7% (n = 1232) at a high risk (**Fig 3B**).

**Fig 3C** shows patients after reclassification based on ProADM cut-off groups and the triage score group. Thereby, “not urgent” and “moderate urgent” patients were upgraded to the next higher category if ProADM levels were >1.5nmol/L. Conversely, “moderate urgent” and “very urgent” patients were downgraded to the next lower category if ProADM levels were ≤0.75nmol/L.

**Fig 4:**
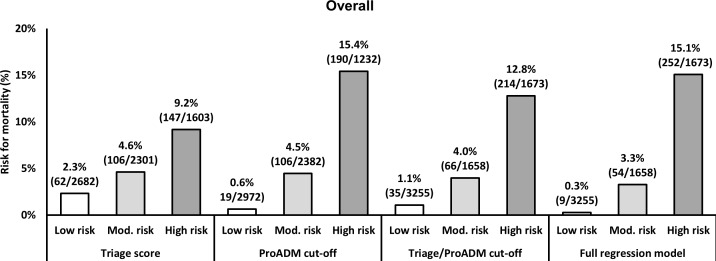
Effect of reclassification on overall identification rate of non-survivors 30 days after emergency department admission. This figure shows mortality in patients classified as “low risk”, “moderate risk” and “high risk” based on the *triage score* only (left panel), the *ProADM cut-offs* only (second from left panel), and the *triage/ProADM cut-offs* combination (second from right panel). Use of the *triage score* only, identified 147/315 (46.7%) non-survivors in the group of patients classified as “high risk”. The combined model however identified 214/315 (67.9%) non-survivors which corresponds to a relative risk increase of 45.6% with the addition of ProADM. Similarly, the number of non-survivors in the low risk “not urgent” population was reduced from 2.3% in the triage score classification to 1.1% in the combined model, resulting in an improvement of 53.5%. ProADM, pro-adrenomedullin.

**Fig 4** shows that the combination of ProADM cut-offs with triage score information thereby resulted in an improvement in sensitivity and specificity with respect to observed mortality. Results were also robust in subgroup analyses based on main medical disciplines or symptoms on ED admission (**S2A–S2G Fig**). These results were also confirmed in category-based reclassification statistics (**Table 4**). The addition of ProADM within cut-off groups resulted in a net reclassification improvement (NRI) of 0.39 for predicting mortality.

**Table 4:** 30-day mortality risk reclassification stratified by survival status.

Triage tool only	Triage tool and ProADM			
	*Not urgent*	*Moderate urgent*	*Very urgent*	Total
***Not urgent***				
**Non-survivors, n**	**29**	**33**	**0**	**62**
**Survivors, n**	**2411**	**209**	**0**	**2620**
**Total patients, n**	**2440**	**242**	**0**	**2682**
**Observed risk (95% CI), %**	**1 (1–2)**	**14 (9–18)**	**-**	**-**
***Moderate urgent***				
**Non-survivors, n**	**6**	**25**	**75**	**106**
**Survivors, n**	**809**	**896**	**490**	**2195**
**Total patients, n**	**815**	**921**	**565**	**2301**
**Observed risk (95% CI), %**	**1 (0–1)**	**3 (2–4)**	**13 (10–16)**	
***Very urgent***				
**Non-survivors, n**	**0**	**8**	**139**	**147**
**Survivors, n**	**0**	**487**	**969**	**1456**
**Total patients, n**	**0**	**495**	**1108**	**1603**
**Observed risk (95% CI), %**	**-**	**2 (1–3)**	**13 (11–14)**	
***Total patients*, *n***				
**Non-survivors**	**35**	**66**	**214**	**315**
**Survivors**	**3220**	**1592**	**1459**	**6271**
**Total**	**3255**	**1658**	**1673**	**6586**

Category-based reclassification for 30-day mortality in patients predicted by the triage tool only against risk predicted a model containing the triage tool and ProADM. The numbers are rounded. ProADM, pro-adrenomedullin. NET Reclassification improvement (NRI): 0.39

Finally, we also investigated the predictive ability of using ProADM and triage scores as continuous variables within a logistic full regression model (right panel of **Fig 4**). In doing so, the proportion of non-survivors in the high-risk category was further increased (n = 252 [80.0% of all non-survivors]), whereas rule out was also improved (9/3255). The relative risk reduction from the simplified to the logistic model was 74.3%. This effect was robust throughout the main medical disciplines with a strong risk reduction in the low risk population and a stable or increased prediction rate in higher risk categories. Stratified for main symptoms and diagnoses on admission, the logistic regression prediction model was superior in ruling out patients than the simplified model. The relative risk reduction ranged between 65.4% and 100%. The performance of the regression model is provided in **S1 File**.

## Discussion

A delayed treatment of patients at risk of adverse outcome, in particular short term mortality, due to suboptimal triage is a daily challenge in emergency centers worldwide [5, 6]. Obviously, there is need for a more accurate and fast ED triage score which stratifies unselected, “real-life” ED patients at the most proximal time point of ED admission. Thereby allowing improved initial management of ED patients. Herein, we found that combining a 3-level triage score (formerly 5-level triage score before simplification) with the prognostic information of ProADM—reflecting different impaired biological pathways (inflammation, imbalanced fluid and electrolyte homeostasis)–resulted in an improved 30-day mortality risk prediction in unselected ED patients. Moreover, combining the triage score and biomarker information resulted in a reclassification of a relevant group of alleged low to intermediate risk patients into the higher risk classes and *vice versa*. Superior to a simplified algorithm, a logistic regression based algorithm adjusted for important confounders revealed higher rule out capacity, a main challenge in ED site of care decisions. If these patients will profit from a prompter therapy regime remains to be investigated in a future interventional study. Thus, our study suggests that the implementation of a combined triage algorithm leads to an increase in the proportion of patients at a high risk of 30-day mortality.

For accurate initial patient triage, several triage scores are known [10, 30, 31]. In this context, we previously validated the performance of the “Manchester Triage Score” (MTS) in an unselected monocentric ED patient cohort. We found a fair prognostic accuracy to predict high initial treatment priority, which is the assigned main focus of a valid triage score [11]. Although not being the primary task of a triage score, the MTS showed only poor performance in 30-day mortality prediction. Even though triage scores are developed to predict high initial treatment priority, daily decisions have to take into account harmful adverse events to further improve early risk stratification. As biomarker, ProADM was shown to provide relevant prognostic information in regard to the risk for adverse events within several prior studies focusing on selected patient subgroups with CAP [32–34], chronic obstructive pulmonary disease (COPD) [15, 35], or cardiovascular diseases [36, 37]. Our study now expands these findings and shows the prognostic value of ProADM for the first time in an unselected patient population.

In “real-life”, triage decisions in ED patients are not always based on clinical risk scores, and are not even evidence- or guideline-based. The fundamental advantages of an improved initial triage include a better resource allocation and avoidance of inadequate use of “fast-track” diagnostics and infrastructure overuse in non-urgent patients. Conversely, patients with real urgencies–initially missed by the triage score alone–might benefit from a prompt treatment. Thus, being aware that modified and improved triage algorithms are needed, this study now fills the gap of lacking large scale studies with high generalizability for unselected ED patients. Beside an improved initial triage using ProADM, such blood biomarkers might additionally provide support in clinical judgment during the ED stay and strengthen the confidence of physicians in making site of care decisions [12].

Using this confounder adjusted combined triage algorithm, we could markedly increase the number of correctly predicted non-surviving patients. Hereby, our data confirms that ProADM improves the triage score for the of 30-day mortality, even in formerly less urgent triage score categories.

We are aware of the currently relatively high costs of ProADM measurement, if used in all consecutive patients on the ED. Nevertheless, based on the fact that 80.0% (versus 46.7% based on triage score only) of non-survivors can be identified, the benefit may outweigh costs associated with marker measurement. At this point, it is unclear whether such an algorithm will result in improved outcomes of patients. There is therefore a need for an interventional trial looking at outcomes and also costs associated with an improved ED triage.

In summary, combining a traditional triage score with clinically relevant ProADM cut-off levels allowed a more objective triage and risk stratification of consecutively entering ED patients, independent of their main complaint or disease. It highly improved prediction of 30-day mortality in lower triage classes which might enforce physician`s compliance with triage decisions. This will likely lead to more overruling decisions in favor of an outpatient (alternatively non-acute care hospital) strategy in subjects without a severe illness. Often, these patients are hospitalized based on a high preference on the part of relatives due to logistical reasons. Adherence to the biomarker enhanced algorithm would also improve patients`safety who formerly would have been discharged home based on a lower triage score but in fact carried a highly significant risk of short-term mortality [38].

Apart from that, a further main strength of this study is the prospective multinational, multicenter design with >6000 unselected representative patients. Thus, these results will be largely generalizable to many international ED settings.

There are some limitations of this study. Firstly, we did not evaluate prediction power of the combined triage algorithm for the primary task of a triage score, the treatment priority. Rather, we investigated a more objective, but not less relevant “hard” endpoint that should be considered in early risk and site of care assessment. Secondly, participating ED personnel were not blinded to the triage score levels, and thus may adapt their priority recommendation accordingly, possibly overestimating the triage score`s performance. Thirdly, we had no validation cohort. Thus, an internal and external validation of the proposed triage approach will be fundamental to prove its effectiveness. Fourthly, within this observational cohort, we were not able to definitively demonstrate whether an improved triage of patients translates into improved outcomes; for this reason, a randomized controlled trial ultimately testing this strong hypothesis is warranted. Fifthly, implementation of the suggested logistic regression based combined algorithm must be available in an electronical form, as required for a pragmatic use. Sixthly, due to missing values, we did not address to smoking and body mass index in the full logistic regression model, as two important confounders of ProADM. However, we included initial diagnoses involving obesity to make a pass at the missing body mass index. Seventhly, we only focused on ProADM based on its performance in previous research [13], but other markers such as high sensitivity C-reactive protein (hsCRP) may also show benefit for 30-day mortality prediction. Herein, we found an area under the receiver operating characteristic curve (ROC AUC) of only 0.70 (data not shown in the results) for CRP compared to 0.83 for ProADM. Even, we did not measure hsCRP, we believe that ROC AUC for hsCRP would be similar to ROC AUC for CRP. Finally, while measurement of most prognostic blood biomarkers (including ProADM) is now commercially available within few hours, fast point-of-care tests are currently being developed that will enable biomarker measurement within minutes [39]. This will definitively improve the pragmatic bedside use of these biomarkers in future trials and clinical practice.

## Conclusion

The combination of established triage scores and ProADM firstly allowed the identification of a higher proportion of ED patients at a high risk of short-term mortality, and secondly improved risk stratification in this heterogeneous patient cohort. Whether the hypothesized benefits will translate into a safe “real-life” improvement of patients`outcome has to be assessed in an interventional trial. Optimized resource allocation will increase patient safety and has high relevance in current health care discussion.

## Supporting Information

S1 Fig Histogram of ProADM. ProADM, pro-adrenomedullin.
(PPTX)

S2 Fig Effect of reclassification on identification rate of non-survivors 30 days after emergency department admission. (A-C) stratified for main medical disciplines on admission, (D-G) stratified for main symptoms on admission. ProADM, pro-adrenomedullin.
(PPTX)

S1 File Coefficients of the logistic regression model for 30-day mortality risk calculation. (A) odds ratios; (B) intercept and calibration slopes
(DOCX)

## Abbreviations

ProADM: proadrenomedullin
ED: emergency department
MTS: Manchester Triage Score
CI: confidence interval
PSI: pneumonia severity index
CAP: community-acquired pneumonia
ICU: intensive care unit
IRB: Institutional Review Boards
SD: standard deviation
IQR: interquartile range
COPD: chronic obstructive pulmonary disease
ROC: receiver operating characteristic
AUC: area under the curve
