# Supplementary figures and images for: The TRIAGE-ProADM Score for an Early Risk Stratification of Medical Patients in the Emergency Department - Development Based on a Multi-National, Prospective, Observational Study
Source: PLoS One. 2016 Dec 22;11(12):e0168076. doi: 10.1371/journal.pone.0168076 (PMC5179054; doi:10.1371/journal.pone.0168076)

## Slide 1
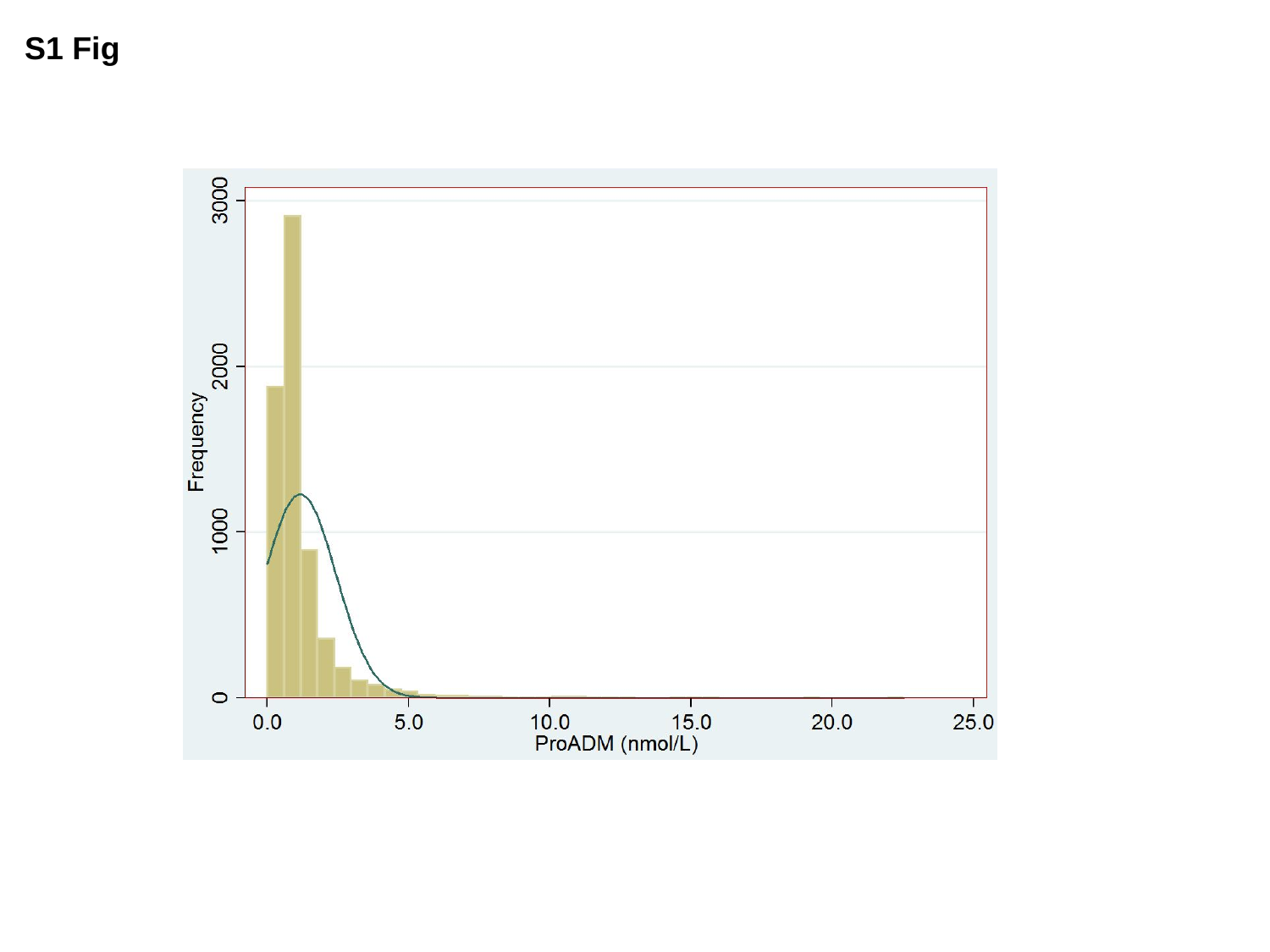

S1 Fig

Supplement: S1 Fig — ProADM, pro-adrenomedullin. (PPTX) [file pone.0168076.s001.pptx]
