# Supplementary material for: The TRIAGE-ProADM Score for an Early Risk Stratification of Medical Patients in the Emergency Department - Development Based on a Multi-National, Prospective, Observational Study
Source: PLoS One. 2016 Dec 22;11(12):e0168076. doi: 10.1371/journal.pone.0168076 (PMC5179054; doi:10.1371/journal.pone.0168076)
Supplement: S2 Fig — (A-C) stratified for main medical disciplines on admission, (D-G) stratified for main symptoms on admission. ProADM, pro-adrenomedullin. (PPTX) [file pone.0168076.s002.pptx]

## Slide 1
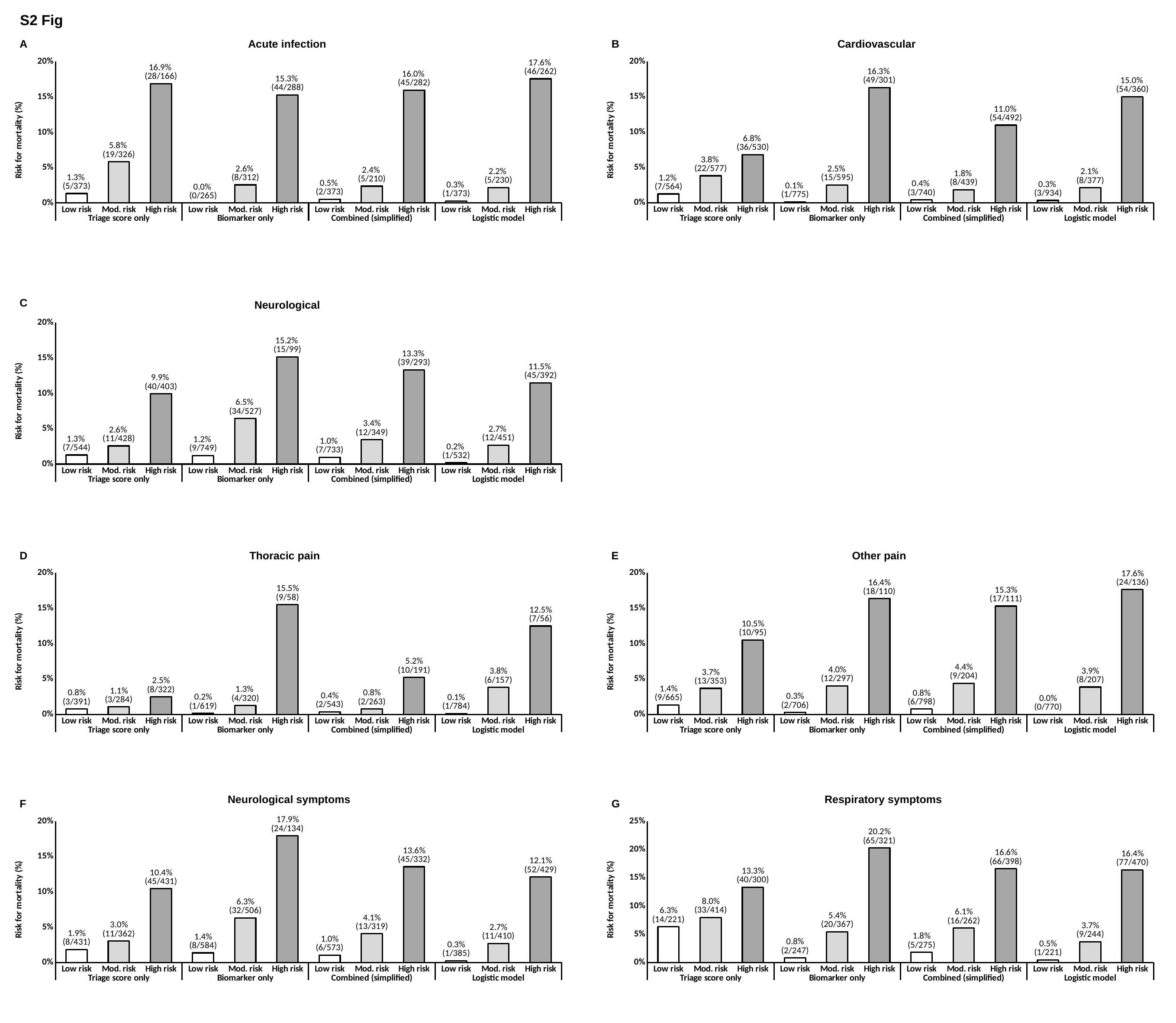

S2 Fig
Cardiovascular
A
Acute infection
B
### Chart
| Category | Non-survivors % |
|---|---|
| Low risk | 0.012411347517730497 |
| Mod. risk | 0.038128249566724434 |
| High risk | 0.06792452830188679 |
| Low risk | 0.0012903225806451613 |
| Mod. risk | 0.025210084033613446 |
| High risk | 0.16279069767441862 |
| Low risk | 0.004054054054054054 |
| Mod. risk | 0.018223234624145785 |
| High risk | 0.10975609756097561 |
| Low risk | 0.0032119914346895075 |
| Mod. risk | 0.021220159151193633 |
| High risk | 0.15 |
### Chart
| Category | Non-survivors % |
|---|---|
| Low risk | 0.013404825737265416 |
| Mod. risk | 0.05828220858895705 |
| High risk | 0.1686746987951807 |
| Low risk | 0.0 |
| Mod. risk | 0.02564102564102564 |
| High risk | 0.1527777777777778 |
| Low risk | 0.005361930294906166 |
| Mod. risk | 0.023809523809523808 |
| High risk | 0.1595744680851064 |
| Low risk | 0.002680965147453083 |
| Mod. risk | 0.021739130434782608 |
| High risk | 0.17557251908396945 |C
Neurological
### Chart
| Category | Non-survivors % |
|---|---|
| Low risk | 0.012867647058823529 |
| Mod. risk | 0.02570093457943925 |
| High risk | 0.09925558312655088 |
| Low risk | 0.012016021361815754 |
| Mod. risk | 0.06451612903225806 |
| High risk | 0.15151515151515152 |
| Low risk | 0.009549795361527967 |
| Mod. risk | 0.034383954154727794 |
| High risk | 0.13310580204778158 |
| Low risk | 0.0018796992481203006 |
| Mod. risk | 0.026607538802660754 |
| High risk | 0.11479591836734694 |D
Thoracic pain
E
Other pain
### Chart
| Category | Non-survivors % |
|---|---|
| Low risk | 0.0076726342710997444 |
| Mod. risk | 0.01056338028169014 |
| High risk | 0.024844720496894408 |
| Low risk | 0.0016155088852988692 |
| Mod. risk | 0.0125 |
| High risk | 0.15517241379310345 |
| Low risk | 0.003683241252302026 |
| Mod. risk | 0.0076045627376425855 |
| High risk | 0.05235602094240838 |
| Low risk | 0.0012755102040816326 |
| Mod. risk | 0.03821656050955414 |
| High risk | 0.125 |
### Chart
| Category | Non-survivors % |
|---|---|
| Low risk | 0.013533834586466165 |
| Mod. risk | 0.036827195467422094 |
| High risk | 0.10526315789473684 |
| Low risk | 0.0028328611898017 |
| Mod. risk | 0.04040404040404041 |
| High risk | 0.16363636363636364 |
| Low risk | 0.007518796992481203 |
| Mod. risk | 0.04411764705882353 |
| High risk | 0.15315315315315314 |
| Low risk | 0.0 |
| Mod. risk | 0.03864734299516908 |
| High risk | 0.17647058823529413 |Neurological symptoms
Respiratory symptoms
F
G
### Chart
| Category | Non-survivors % |
|---|---|
| Low risk | 0.018561484918793503 |
| Mod. risk | 0.03038674033149171 |
| High risk | 0.10440835266821345 |
| Low risk | 0.0136986301369863 |
| Mod. risk | 0.06324110671936758 |
| High risk | 0.1791044776119403 |
| Low risk | 0.010471204188481676 |
| Mod. risk | 0.04075235109717868 |
| High risk | 0.1355421686746988 |
| Low risk | 0.0025974025974025974 |
| Mod. risk | 0.026829268292682926 |
| High risk | 0.12121212121212122 |
### Chart
| Category | Non-survivors % |
|---|---|
| Low risk | 0.06334841628959276 |
| Mod. risk | 0.07971014492753623 |
| High risk | 0.13333333333333333 |
| Low risk | 0.008097165991902834 |
| Mod. risk | 0.05449591280653951 |
| High risk | 0.20249221183800623 |
| Low risk | 0.01818181818181818 |
| Mod. risk | 0.061068702290076333 |
| High risk | 0.1658291457286432 |
| Low risk | 0.004524886877828055 |
| Mod. risk | 0.036885245901639344 |
| High risk | 0.16382978723404254 |
