# Supplementary material for: The TRIAGE-ProADM Score for an Early Risk Stratification of Medical Patients in the Emergency Department - Development Based on a Multi-National, Prospective, Observational Study
Source: PLoS One. 2016 Dec 22;11(12):e0168076. doi: 10.1371/journal.pone.0168076 (PMC5179054; doi:10.1371/journal.pone.0168076)
Supplement: S1 File — (A) odds ratios; (B) intercept and calibration slopes (DOCX) [file pone.0168076.s003.docx]

**S1 File**

**Coefficients of the logistic regression model for 30-day mortality risk calculation**

**A**

**Odds ratios**

Figure 1. TRIAGE: 1, “blue”, 2, “green”, 3, “yellow”, 4, “orange”, 5, “red”; HAUPT_SY: main symptoms on admission, 1, “other pain”, 2, “thoracic pain”, 3, “neurological symptoms”, 4, “respiratory symptoms”, 5, “general worsening”, 8, “blood loss”, 9, “diarrhea, vomitus, dysuria”, 10, “fever”, 11, “other”; HAUPT_DG_initial: main medical discipline on admission, 1, “Acute infection”, 2, “cardiovascular”, 3, “metabolic”, 4, “cancer”, 5, “neurological”, 6, “gastrointestinal”, 7, “pulmonary”, 8, “other”.

**B**

**Intercept and calibration slopes**

Figure 2. TRIAGE: 1, “blue”, 2, “green”, 3, “yellow”, 4, “orange”, 5, “red”; HAUPT_SY: main symptoms on admission, 1, “other pain”, 2, “thoracic pain”, 3, “neurological symptoms”, 4, “respiratory symptoms”, 5, “general worsening”, 8, “blood loss”, 9, “diarrhea, vomitus, dysuria”, 10, “fever”, 11, “other”; HAUPT_DG_initial: main medical discipline on admission, 1, “Acute infection”, 2, “cardiovascular”, 3, “metabolic”, 4, “cancer”, 5, “neurological”, 6, “gastrointestinal”, 7, “pulmonary”, 8, “other”.
